# Does Authentic Leadership Foster Work Engagement? A Post-Pandemic Systematic Review and Meta-Analysis

**DOI:** 10.12688/f1000research.180597.1

**Published:** 2026-05-19

**Authors:** S. Daniel Kristanto, Sylvia Permata Sari, Ardi Ardi

**Affiliations:** 1 Magister Management, Faculty of Economics and Business, Pelita Harapan University, Tangerang, Banten, 15811, Indonesia; 2 Faculty of Economics and Business, Pelita Harapan University, Tangerang, Banten, 15811, Indonesia

**Keywords:** Authentic Leadership, Work Engagement, Meta-Analysis, Job Demands-Resources Model, Social Exchange Theory, Post-Pandemic, Random-Effects Model, SDG 8

## Abstract

**Background:**

Work engagement has emerged as a critical psychological resource for organizational resilience in the post-pandemic era. Authentic leadership is theoretically positioned as a key antecedent of engagement, yet empirical findings regarding the strength and consistency of this relationship remain fragmented, particularly across emerging economies. This study synthesizes post-pandemic evidence on the authentic leadership and work engagement relationship, integrating Social Exchange Theory and the Job Demands-Resources model as complementary interpretive frameworks.

**Methods:**

Following PRISMA 2020 guidelines, a systematic search was conducted across SCOPUS, Web of Science, Wiley Online, and APA PsycNet for peer-reviewed empirical studies published between 2020 and 2025. Only studies reporting bivariate Pearson correlation coefficients were included. Nineteen studies spanning 12 countries across Asia, Europe, Africa, South America, and Oceania met the inclusion criteria, yielding a combined sample of 6,916 participants. A random-effects meta-analysis using Restricted Maximum Likelihood estimation with Knapp-Hartung adjustment was performed. Subgroup analyses examined cultural power distance, industry type, organizational sector, and country classification as moderators.

**Results:**

The analysis revealed a significant positive pooled correlation between authentic leadership and work engagement ( *r* = 0.41, 95% *CI* [0.35, 0.47], *p* < .001), classified as a medium effect, with substantial heterogeneity ( *I ^2^* = 87.20%). None of the four tested moderators significantly explained between-study variance, although limited statistical power due to unbalanced subgroup sizes precludes definitive conclusions regarding contextual invariance. Robustness was supported by a Fail-Safe N of 7,783 and sensitivity analyses. Narrative synthesis identified psychological capital and psychological empowerment as the most consistently supported mediating mechanisms, with organizational performance, innovative work behavior, and leadership aspirations as preliminary downstream outcomes.

**Conclusions:**

Authentic leadership demonstrated a consistent positive association with work engagement across the diverse contexts represented in the included studies. These findings suggest that authentic leadership development may contribute to fostering decent work environments, with relevance to Sustainable Development Goal 8.

## 1. Introduction

In today’s volatile and rapidly evolving business environment, work engagement has emerged as a critical psychological resource for organizational survival and competitive advantage ( Towsen et al., 2020). Work engagement is a positive work-related state of mind characterized by vigor, dedication, and absorption ( Farid et al., 2022; Koon & Ho, 2021). This psychological state enables employees to cope with elevated job demands and sustain mental resilience ( Decuypere & Schaufeli, 2021; Towsen et al., 2020). Recent empirical research evidence indicates that engaged employees demonstrate elevated innovative behavior and enhanced performance, including reduced turnover intentions, which are essential characteristics for organizations functioning in the post-pandemic new normal ( Bai et al., 2022; Towsen et al., 2020). A lack of engagement is increasingly associated with psychological withdrawal and attrition, particularly among younger demographics such as Generation Z entering high-pressure industries like hospitality and manufacturing ( Sigaeva et al., 2022). Consequently, organizations are increasingly seeking leadership models that can cultivate this vital psychological resource.

Among various leadership styles, authentic leadership has attracted considerable academic interest as a significant antecedent of work engagement. Based on the foundational work of Walumbwa et al. (2008), authentic leadership is conceptualized as a pattern of leader behavior that draws upon positive psychological capacities and a positive ethical climate to foster greater self-awareness, an internalized moral perspective, balanced processing of information, and relational transparency ( Silva et al., 2023; Vermeulen & Scheepers, 2020). Theoretical frameworks such as Social Exchange Theory (SET) and the Job Demands-Resources (JD-R) model have been used to explain workforce dynamics including authentic leadership and work engagement relationship ( Farid et al., 2022; Li et al., 2025; Pulido-Martos et al., 2023). From a SET perspective, authentic leaders exhibiting transparency and moral integrity create a norm of reciprocity, prompting followers to return the leader’s support with heightened dedication and involvement ( Farid et al., 2022; Vermeulen & Scheepers, 2020). The JD-R model posits that authentic leadership functions as a crucial job resource that mitigates occupational stress and enhances motivation, thereby improving engagement either directly or through intermediaries such as psychological capital and well-being ( Lopez-Zafra et al., 2022; Pulido-Martos et al., 2023; Silva et al., 2023).

Despite the theoretical soundness of this link, empirical findings regarding its strength and consistency remain fragmented, particularly in the post-pandemic era ( Macamo & Klasmeier, 2024). Prior meta-analyses reported positive correlations based largely on pre-pandemic data drawn from Western contexts ( Decuypere & Schaufeli, 2021). More recent studies conducted in emerging economies have produced considerably heterogeneous effects. Research conducted in China’s manufacturing sector has found moderate correlations ( *r* = 0.26) between authentic leadership and engagement ( Bai et al., 2022), whereas studies in Malaysia and South Africa’s service sectors demonstrate considerably stronger effects, with correlations surpassing 0.50 ( Koon & Ho, 2021; Vermeulen & Scheepers, 2020). This substantial variation raises an important question: under what conditions is authentic leadership most effective in fostering engagement? Recent research further emphasizes that leader behavioral consistency is pivotal, as inconsistency can diminish the beneficial impacts on follower well-being ( Macamo & Klasmeier, 2024). These patterns suggest that the effect of authentic leadership may be contingent on contextual factors, such as cultural or industrial environments, that prior meta-analyses may have overlooked.

A further methodological gap exists in prior syntheses. Previous meta-analyses have pooled bivariate Pearson correlation coefficients ( *r*) together with effect sizes estimated from regression ( *β*) coefficients, a practice that introduces estimation bias and compromises the accuracy of the pooled effect size ( Paul & Barari, 2022). A rigorous synthesis is required that strictly uses bivariate Pearson correlation coefficients to obtain an accurate estimate of the population effect size ( Xiao, 2024). In addition, the global disruption experienced in 2020–2022 triggered fundamental transformations in work organization that require renewed examination of the relationship between leadership and engagement, especially in the regions that remain under-represented in the leadership and engagement literature, such as Southeast Asia, Eastern Europe, and Africa ( Niswaty et al., 2021; Sigaeva et al., 2022; Vermeulen & Scheepers, 2020).

To address these gaps, this study integrates quantitative meta-analysis and qualitative narrative synthesis to pursue four research questions:

• RQ1: What is the pooled effect size of the relationship between authentic leadership and work engagement in empirical studies published from 2020 to 2025?
• RQ2: Do cultural, industrial, sectoral, and country-level contexts moderate this relationship?
• RQ3: How can the observed relationship between authentic leadership and work engagement be interpreted through the complementary lenses of SET and the JD-R model?
• RQ4: What mediating mechanisms and organizational outcomes emerge from the included studies?

Beyond its theoretical contributions, this study has practical relevance to promoting decent work environments, as articulated in the United Nations Sustainable Development Goal 8 (SDG 8: Decent Work and Economic Growth). If authentic leadership consistently fosters work engagement across diverse organizational contexts, then leadership development programs targeting authenticity may serve as a scalable mechanism for advancing SDG 8 targets, particularly in emerging economies where both engaged workforces and effective leadership remain critical development priorities.

**Figure 1.**
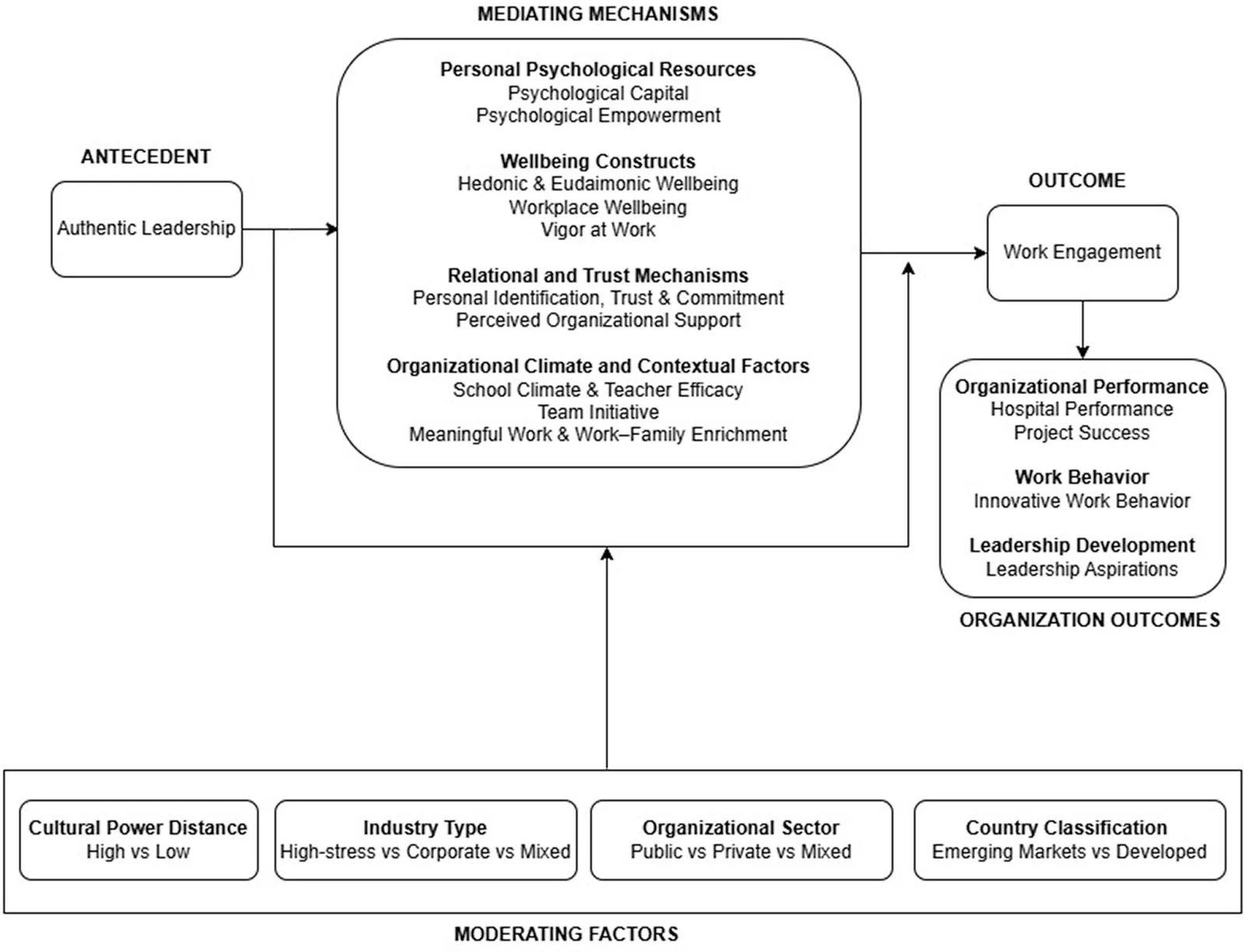
An integrated research model in this study. An integrated research model is used in this study. Authentic leadership is the antecedent and work engagement is the central psychological outcome. Several mediating mechanisms were identified in the pathway between them and the organizational outcomes. The four contextual moderators are cultural power distance, industry type, organizational sector, and country classification.

Figure 1 presents the integrated research model guiding this investigation. The model positions authentic leadership as the antecedent and work engagement as the central psychological outcome, with mediating mechanisms explaining the pathway between them. The model further extends to distal organizational outcomes and incorporates four contextual moderators: cultural power distance, industry type, organizational sector, and country classification.

## 2. Theoretical background and hypothesis development

### 2.1 Authentic leadership

Authentic leadership comprises four interrelated dimensions: self-awareness, relational transparency, balanced processing, and an internalized moral perspective ( Walumbwa et al., 2008). Self-awareness refers to leaders’ understanding of their own strengths, weaknesses, and the way they derive meaning from the world ( Silva et al., 2023; Walumbwa et al., 2008). Balanced processing describes the objective analysis of relevant data prior to decision-making ( Silva et al., 2023; Walumbwa et al., 2008). Relational transparency involves openly sharing one’s thoughts and feelings, thereby fostering trust within leader–follower dyads ( Silva et al., 2023; Walumbwa et al., 2008). The internalized moral perspective reflects decision-making and behavior guided by internal ethical standards rather than external pressures ( Almutairi et al., 2025; Sigaeva et al., 2022; Silva et al., 2023). These four dimensions collectively distinguish authentic leadership from related constructs such as transformational and servant leadership, as they emphasize the leader’s psychological integrity rather than specific behavioral repertoires ( Srimongkolkul et al., 2025). Empirical evidence confirms that these genuine behaviors foster trust in leadership, which in turn enhances employee well-being and engagement ( Baquero, 2023). Leaders who exhibit this style are particularly well-suited to cultivating a positive organizational climate, a factor increasingly recognized as essential for navigating the dynamic post-pandemic working environment ( Khan et al., 2021; Koon & Ho, 2021).

### 2.2 Work engagement

Work engagement is defined as “a positive, fulfilling, work-related state of mind that is characterized by vigor, dedication, and absorption” ( Schaufeli et al., 2002). Vigor reflects high levels of energy and mental resilience while working, along with a willingness to invest effort and persist in the face of difficulties ( Koon & Ho, 2021; Lopez-Zafra et al., 2022; Schaufeli et al., 2002). Dedication refers to a sense of significance, enthusiasm, pride, and challenge derived from one’s work ( Koon & Ho, 2021; Schaufeli et al., 2002). Absorption denotes a state of being fully concentrated and engrossed in one’s tasks ( Koon & Ho, 2021; Lopez-Zafra et al., 2022). Engaged employees are more likely to exhibit creative problem-solving, innovative work behavior, and reduced turnover intentions, outcomes that enhance organizational competitive advantage ( Aldabbas et al., 2025; Arokiasamy et al., 2022; Shao et al., 2025). Given these outcomes, understanding the antecedent conditions that foster engagement remains a central concern in organizational behavior research.

### 2.3 Theoretical framework

The relationship between authentic leadership and work engagement can be explained by integrating two complementary theoretical perspectives: the JD-R model and SET ( Farid et al., 2022; Li et al., 2025).

The JD-R model distinguishes between job demands, aspects of work that require sustained physical or psychological effort, such as time pressure and workload, and job resources, aspects that facilitate goal achievement, reduce demands, and stimulate personal growth, such as autonomy, social support, and leadership ( Li et al., 2025; Niswaty et al., 2021; Pulido-Martos et al., 2023; Silva et al., 2023). Within this framework, authentic leadership operates as a critical job resource: leaders who demonstrate transparency and balanced processing reduce uncertainty and role ambiguity, thereby lowering psychological demands while simultaneously fostering the conditions for intrinsic motivation and engagement ( Lopez-Zafra et al., 2022).

SET complements this motivational perspective with a relational lens. The theory posits that social interactions within organizations are governed by norms of reciprocity ( Farid et al., 2022; Khan et al., 2021). When leaders demonstrate fairness, transparency, and moral integrity, followers perceive these behaviors as high-quality social investments. This perception generates a felt obligation to reciprocate through heightened dedication, vigor, and absorption, the core dimensions of work engagement ( Towsen et al., 2020). The reciprocity dynamic is particularly relevant because it explains why authentic leadership effects may extend beyond the instrumental provision of resources to encompass relational trust and affective commitment ( Lux et al., 2023).

Together, the JD-R model and SET provide a comprehensive framework: the JD-R model explains how authentic leadership activates engagement through resource provision, while SET explains why followers respond to such leadership with enhanced engagement through relational reciprocity. This integrative lens underpins the hypotheses developed below.

### 2.4 Hypothesis development

**2.4.1 Main effect hypothesis**

Building on the theoretical integration outlined above, the first hypothesis concerns the overall direction and significance of the authentic leadership–work engagement relationship. Empirical studies published between 2020 and 2025 have consistently reported a positive association between the two constructs, although the magnitude of the association varies across samples. In the Russian hospitality sector, Sigaeva et al. (2022) found that authentic leaders substantially augment the psychological capital of Generation Z employees, thereby fostering engagement. Shao et al. (2025) demonstrated that authentic leadership enhances teacher engagement in China by fostering a supportive school environment. Research conducted in high-context cultures, including Indonesia and Pakistan, has confirmed that authentic leaders promote trust and well-being, which serve as preconditions for engagement ( Farid et al., 2022; Niswaty et al., 2021). This consistency is observed across geographically diverse contexts, from South Africa ( Towsen et al., 2020) to Spain ( Lopez-Zafra et al., 2022), Portugal ( Silva et al., 2023), and Uruguay ( Cozzo Cabrera et al., 2025). Accordingly, the following hypothesis is proposed:

H1: Authentic leadership is significantly and positively correlated with work engagement in post-pandemic organizational contexts.

**2.4.2 Contextual moderators**

While the overall relationship between authentic leadership and work engagement is expected to be positive, the magnitude of this effect may not be uniform across all settings ( Bai et al., 2022; Macamo & Klasmeier, 2024). The substantial variation observed among studies conducted in emerging economies suggests that authentic leadership effects may be moderated by contextual boundary conditions ( Niswaty et al., 2021). This study examines four moderators derived from theoretical reasoning and empirical observations. Although theoretical arguments can be advanced for specific directional expectations, as elaborated for each moderator below, the limited and sometimes contradictory empirical evidence across post-pandemic studies, combined with the exploratory nature of cross-cultural moderation testing with a relatively small number of studies, warrants non-directional hypothesis formulation ( Hansen et al., 2022). The following four moderation hypotheses are therefore stated in non-directional form.

Hofstede’s cultural dimensions framework provides a theoretical basis for expecting cross-cultural variation. In high power distance cultures, characteristic of many Asian, Middle Eastern, and Eastern European societies, leaders are traditionally perceived as hierarchically distant. In such contexts, authentic leader behaviors, such as relational transparency and balanced processing, constitute a comparatively rare and valuable organizational resource. From a SET perspective, the openness displayed by authentic leaders in high power distance cultures may generate a deeper sense of trust and psychological safety than in low power distance or individualistic contexts, where such behaviors are more normatively expected. The resulting obligation to reciprocate with engagement may therefore be stronger in high power-distance environments ( Assi et al., 2024; Farid et al., 2022). Conversely, in low power-distance or individualistic cultures, authentic leadership may primarily serve as a motivational job resource that facilitates autonomy and work meaningfulness, thereby enhancing intrinsic motivation through the JD-R pathway ( Lopez-Zafra et al., 2022; Zheng et al., 2025). This cross-cultural differentiation suggests that although the direction of the authentic leadership and engagement relationship is expected to be consistently positive, its strength may vary systematically across cultural contexts. Accordingly:

H2a: The strength of the correlation between authentic leadership and work engagement varies significantly across different cultural power distance contexts.

The JD-R model provides a basis for anticipating industry-based moderation. Different industries impose distinct job demands and therefore require specific investments in resources. In high-stress or human-service industries, such as healthcare, education, and hospitality, employees are subjected to considerable emotional labor and psychological strain. In these settings, authentic leadership may be particularly effective as a protective resource against burnout by establishing ethical boundaries and providing emotional support. Empirical evidence from the Russian hospitality industry ( Sigaeva et al., 2022) and the Chinese education system ( Shao et al., 2025) demonstrates that authentic leadership significantly enhances follower psychological capital in such contexts. In contrast, in more technically oriented corporate sectors, authentic leadership’s influence may primarily operate through role clarity and objective decision-making processes rather than emotional support. Given that authentic leadership emphasizes holistic human development, its engagement-enhancing effects are expected to be most pronounced in industries that rely heavily on human interaction and psychological resilience. Therefore:

H2b: The strength of the correlation between authentic leadership and work engagement varies significantly across industry types.

Public and private sector organizations operate under different governance structures and normative frameworks, which may shape how authentic leadership influences engagement. Public sector employees frequently operate within bureaucratic systems characterized by rules and procedural constraints that can limit individual autonomy. In this environment, authentic leaders may generate engagement by creating psychological safety and fostering openness within otherwise rigid organizational structures. In the private sector, which is generally more market-oriented and performance-driven, authentic leadership may operate as a strategic resource for fostering innovation and high-quality social exchange relationships that encourage employees to invest fully in their roles. While both sectors stand to benefit from authentic leadership, the differing levels of organizational control and reward systems suggest variability in the magnitude of the leadership-engagement nexus. Accordingly:

H2c: The strength of the correlation between authentic leadership and work engagement varies significantly between organizational sectors.

The effectiveness of authentic leadership in fostering work engagement may also vary across different economic development contexts. Organizations in emerging and developed economies operate within fundamentally different institutional frameworks. Emerging economies are generally characterized by weaker regulatory stability, greater economic uncertainty, less formalized labor markets, and developing governance structures, whereas developed economies feature stronger institutional frameworks, more stable economic conditions, and well-established organizational support systems. From a SET perspective, the reciprocity dynamic underlying the authentic leadership-work engagement relationship may be more pronounced in emerging economies, where authentic leadership is a comparatively scarce yet highly valued organizational resource. Employees who perceive their leaders as genuinely authentic in environments where such leadership is less institutionally guaranteed may respond with proportionally stronger engagement as a form of reciprocation. Therefore:

H2d: The strength of the correlation between authentic leadership and work engagement varies significantly between emerging markets and developed countries.

## 3. Methods

### 3.1 Research design and search strategy

This study employed a systematic review and meta-analysis approach in accordance with the Preferred Reporting Items for Systematic Reviews and Meta-Analyses (PRISMA) 2020 framework ( Page et al., 2021). The process comprised three sequential phases: systematic literature searching, screening and selection based on predefined criteria, and quantitative synthesis via meta-analysis.

The literature search was conducted on March 6 ^th^, 2026, across four databases: SCOPUS, Web of Science, Wiley Online, and APA PsycNet. The following keyword combination was applied across all databases: (“Authentic leadership”) AND ((“employee” OR “work”) AND “engagement”). To enhance the precision of the search results, the following filter criteria were applied: (1) year of publication: 2020–2025; (2) document type: article; (3) publication stage: final; (4) source type: journal; (5) language: English; and (6) access type: open access. The open-access restriction was applied to ensure full-text accessibility for both reviewers during the screening process and for readers seeking to verify or replicate the systematic search. This decision aligns with emerging meta-analytic practice that prioritizes transparency and reproducibility ( Page et al., 2021). However, it is acknowledged that this filter may have excluded relevant studies published in subscription-based journals, and the implications of this restriction for the representativeness of the evidence base are discussed in the limitations section (Section 5.7).

The 2020–2025 publication window was selected to capture the body of empirical research produced during and in the aftermath of the COVID-19 pandemic, which fundamentally reshaped organizational contexts and leadership dynamics worldwide ( Farid et al., 2022). It should be noted that this criterion reflects publication year rather than the timing of data collection. Some studies published in 2020 or 2021 may have collected data prior to or during the early stages of the pandemic. As such, the term ‘post-pandemic’ is used throughout this manuscript to refer to the period of scholarly production rather than to assert that all primary data were collected under post-pandemic conditions.

### 3.2 Inclusion and exclusion criteria

A screening protocol was developed to select studies suitable for quantitative synthesis. Studies were included if they met the following criteria: (1) published in a peer-reviewed journal; (2) examined a direct empirical relationship between authentic leadership and work engagement; and (3) reported the Pearson correlation coefficient ( *r*) explicitly. The requirement for bivariate Pearson correlations was imposed to maintain consistency in effect size estimation. As noted by Paul and Barari (2022), pooling correlation coefficients with regression-derived beta ( *β*) values introduces estimation bias because regression coefficients are subject to multicollinearity and model specification, rendering them non-comparable as bivariate effect sizes ( Xiao, 2024). Studies were therefore excluded if they: (1) were not peer-reviewed (e.g., conference proceedings, book chapters, dissertations); (2) utilized qualitative or theoretical methods without empirical data; or (3) reported only regression coefficients ( *β*) without an accompanying correlation matrix.

### 3.3 Study selection process

**Figure 2.**
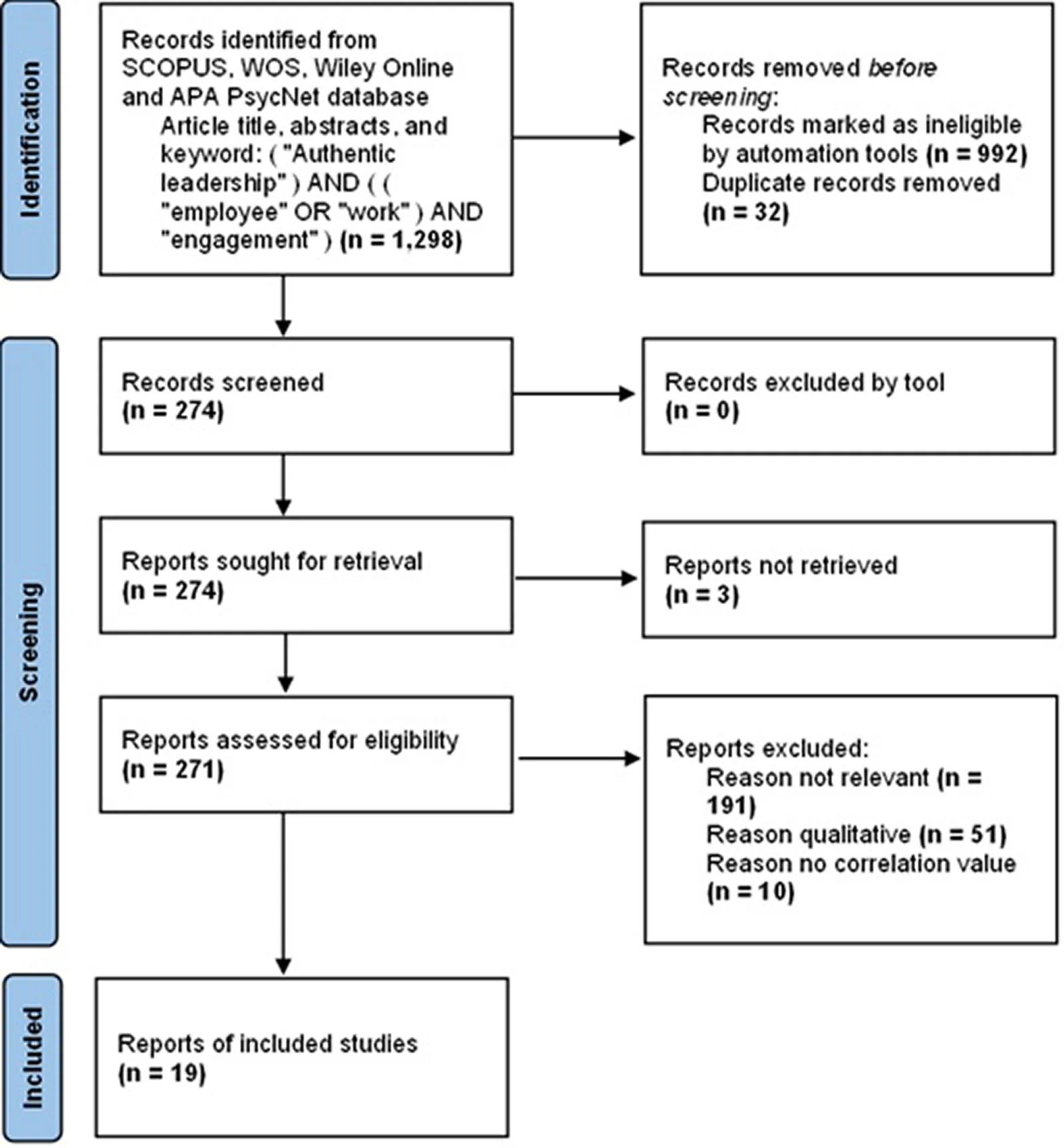
PRISMA 2020 Flow used in this study. Step by step, following PRISMA 2020, until 19 studies have been identified.

Two independent reviewers screened all records at the title/abstract and full-text stages. Any disagreements were resolved through discussion until consensus was achieved. The PRISMA 2020 flow diagram ( Figure 2; also available in Kristanto, S. D. (2026a)) illustrates the step-by-step selection process. The initial database search identified 1,298 records. Prior to screening, 992 records were removed as ineligible based on the applied filter criteria (document type, open access, source type, and language), and 32 duplicate records were removed across databases. The remaining 274 records were screened for relevance by title and abstract. All 274 records proceeded to the retrieval stage; however, three reports could not be retrieved, leaving 271 reports for full-text eligibility assessment.

After a detailed full-text assessment, 252 reports were excluded for the following reasons: lack of relevance to the authentic leadership-work engagement relationship (n = 191), qualitative or theoretical design without empirical data (n = 51), and absence of Pearson correlation values (n = 10). This systematic process yielded a final set of 19 studies ( *k* = 19) included in the meta-analysis, representing a total sample size of 6,916 participants across 12 countries spanning Asia (China, Indonesia, Jordan, Malaysia, Pakistan), Australasia (Australia), Europe (Germany, Portugal, Russia, Spain), South America (Uruguay), and Africa (South Africa). The characteristics of the 19 included studies, including titles, authors, publication year, journal name, publisher, country, sector/industry, sample size, and reported correlation value, are summarized in Table 1.

**Table 1.** Characteristics of the 19 included studies.

No	Titles	Author(s) & Year	Journal Name	Publisher	Country	Sector / Industry	Sample Size ( *N*)	Correlation ( *r*)
1	Linking authentic leadership and management capability to public hospital performance: the role of work engagement	Aboramadan et al. (2021)	International Journal of Organizational Analysis	Emerald Publishing Limited	Jordan	Healthcare (Public Hospitals)	380	0.35
2	Nurse Managers Authentic Leadership and their Relationship with Work Engagement among Registered Nurses	Assi et al. (2024)	Nursing Forum	John Wiley & Sons Ltd	Jordan	Healthcare (Public Hospitals)	238	0.37
3	The Impact of Authentic Leadership on Innovative Work Behavior: Mediating Roles of Proactive Personality and Employee Engagement	Bai et al. (2022)	Frontiers in Psychology	Frontiers Media S.A.	China	Manufacturing (High-tech)	311	0.26
4	Leadership aspirations: The roles of authentic leadership and work engagement	Cozzo Cabrera et al. (2025)	Revista Latinoamericana de Psicología	Fundación Universitaria Konrad Lorenz	Uruguay	Military	320	0.45
5	“Doing good and feeling good” Relationship between authentic leadership with followers’ work engagement: The mediating role of hedonic and eudaimonic wellbeing	Farid et al. (2022)	Frontiers in Public Health	Frontiers Media S.A.	Pakistan	Telecommunication	250	0.42
6	Unwrapping Software Projects Success in Asia: Assessing the Role Of Authentic Leadership, Psychological Empowerment, and Job Engagement in Project Success Using a Serial-Mediation Approach	Khattak et al. (2022)	SAGE Open	SAGE	Pakistan	Software Industry	343	0.5
7	Authentic leadership and employee engagement: The role of employee well-being	Koon & Ho (2021)	Human Systems Management	IOS Press	Malaysia	Service (SMEs)	150	0.75
8	Initiative in Work Teams: Lever between Authentic Leadership and Results	Lisbona et al. (2021)	International Journal of Environmental Research and Public Health	MDPI	Spain	Various (SME and Large)	310	0.51
9	Vigor at work mediates the effect of transformational and authentic leadership on engagement	Lopez-Zafra et al. (2022)	Scientific Reports	Nature Portfolio (Springer Nature)	Spain	Various (Private & Public)	215	0.34
10	Reframing commitment in authentic leadership: Untangling relationship-outcome processes	Lux et al. (2023)	Journal of Management & Organization	Cambridge University Press	Australia	Various	281	0.38
11	Authentic leadership - for better and for worse? Leader well-being and inconsistency as moderating factors in the relation between daily authentic leadership and follower well-being	Macamo & Klasmeier (2024)	European Journal of Work and Organizational Psychology	Routledge (Taylor & Francis Group)	Germany	Various (Industry, Finance, etc.)	161	0.31
12	Investigating the effect of authentic leadership and employees’ psychological capital on work engagement: evidence from Indonesia	Niswaty et al. (2021)	Heliyon	Elsevier Ltd	Indonesia	Public Service	192	0.39
13	Authentic leadership and personal and job demands/resources: A person-centered approach and links with work-related subjective well-being	Pulido-Martos et al. (2023)	Current Psychology	Springer	Spain	Various (Public and Private Sector)	968	0.3
14	The impact of authentic leadership on the work engagement of primary and secondary school teachers: The serial mediation role of school climate and teacher efficacy	Shao et al. (2025)	PLoS One	Public Library of Science (PLOS)	China	Education	1,043	0.3
15	In Search of Effective Gen Z Engagement in the Hospitality Industry: Revisiting Issues of Servant and Authentic Leadership	Sigaeva et al. (2022)	Sustainability	MDPI	Russia	Hospitality	393	0.28
16	How Does Authentic Leadership Boost Work Engagement? Exploring the Mediating Role of Work Meaningfulness and Work-Family Enrichment	Silva et al. (2023)	Administrative Sciences	MDPI	Portugal	Various (Service, etc.)	292	0.48
17	The Relationship Between Authentic Leadership, Psychological Empowerment, Role Clarity, and Work Engagement: Evidence From South Africa	Towsen et al. (2020)	Frontiers in Psychology	Frontiers Media S.A.	South Africa	Mining (Coal)	236	0.49
18	Mediating effect of perceived organisational support on authentic leadership and work engagement	Vermeulen & Scheepers (2020)	SA Journal of Human Resource Management	AOSIS	South Africa	Information Technology	162	0.51
19	Relationship between work engagement and healthy work environment among Chinese ICU nurses: The mediating role of psychological capital	Xue et al. (2023)	Nursing Open	John Wiley & Sons Ltd	China	Healthcare	671	0.34

Full list of the 19 included studies, including titles, authors, publication year, journal name, publisher, country, sector/industry, sample size, and reported correlation value.

### 3.4 Quality assessment

The methodological quality of the 19 included studies was evaluated using the Mixed Methods Appraisal Tool (MMAT) version 2018 ( Hong et al., 2018). This tool was selected for its ability to appraise diverse study designs, including quantitative and mixed-methods research. Following the MMAT protocol, each study was first screened against two prerequisite questions regarding the clarity of research objectives and the adequacy of the data to address them. The 18 quantitative studies were subsequently assessed using the Quantitative Non-Randomized category (Section 3 of the MMAT), which evaluates five criteria: sampling representativeness, measurement validity, data completeness, control for confounding variables, and appropriate exposure measurement. One mixed-methods study was further assessed using the Mixed Methods category (Section 5 of the MMAT) to evaluate the rationale for integration and coherence between qualitative and quantitative components.

Quality assessment was performed independently by two reviewers. Any discrepancies in appraisal scores were resolved through discussion until a consensus was reached. Following MMAT guidelines, studies were not excluded based on quality scores; instead, the assessment was used to inform the discussion regarding the strength of the evidence linking authentic leadership to work engagement.

### 3.5 Data analysis

The meta-analysis was conducted using JASP software (Version 0.96.0) to calculate effect sizes and generate graphical outputs ( Ashour, 2024). A random-effects model with the Restricted Maximum Likelihood (REML) estimation method was employed to account for anticipated heterogeneity across industries and cultural contexts ( Partlett & Riley, 2017). All reported Pearson correlation coefficients ( *r*) were first transformed to Fisher’s *z* scale for analysis and subsequently back-transformed to *r* for reporting purposes ( Xiao, 2024). The magnitude of pooled effect sizes was interpreted using Cohen’s conventional benchmarks: small (r < 0.30), medium (0.30 ≤ r < 0.50), and large (r ≥ 0.50) ( Cohen, 2013; Xiao, 2024). To provide contextual anchoring beyond these generic thresholds, the pooled estimate was additionally evaluated against the most recent comparable meta-analysis on authentic leadership and work engagement ( Decuypere & Schaufeli, 2021).

Heterogeneity across studies was evaluated using Cochran’s *Q*-statistic and the *I ^2^* index, with *I ^2^* values exceeding 75% indicating substantial heterogeneity ( Xiao, 2024). The robustness of the pooled estimate was assessed using 95% Confidence Intervals ( *CI*) and 95% Prediction Intervals ( *PI*). Publication bias was evaluated through Funnel Plot visual inspection, Egger’s weighted regression test, and Rosenthal’s Fail-Safe N. In sensitivity analysis, the Baujat plot was employed to identify individual study contributions to overall heterogeneity and their influence on the pooled effect size estimate. This study was conducted according to the PRISMA guidelines available in Kristanto, S. D. (2026e) and Kristanto, S. D. (2026f ), and all software code is available in the Zenodo repository: Kristanto, S. D. (2026d).

### 3.6 Moderation analysis

Categorical subgroup analyses were conducted to test the moderation hypotheses (H2a–H2d). Four moderator variables were operationalized as follows, with study-level classifications summarized in Table 2. Cultural power distance (H2a): Each study was classified as high or low power distance based on the Hofstede Power Distance Index (PDI) score of the study’s country of origin ( Hofstede, 2001). A threshold of PDI = 50 was employed, consistent with the median split approach commonly used in other studies, where PDI > 50 = high power distance and PDI ≤ 50 = low power distance ( Ulmer et al., 2018). This yielded 15 studies in the high-power-distance category and 4 in the low-power-distance category. It is acknowledged that one country (South Africa, PDI = 49) falls at the boundary of this classification; the implications of this borderline case are considered in the limitations section.

**Table 2.** Subgroup categories in meta-analysis.

No	Author & Year	Country	Country Classification	Country Power Distance Score	Culture (Power Distance)	Industry Type	Sector	Sample Size (N)	Correlation (r)
1	**Aboramadan et al. (2021)**	Jordan	Emerging	70	High	High-stress	Public	380	0.35
2	**Assi et al. (2024)**	Jordan	Emerging	70	High	High-stress	Public	238	0.37
3	**Bai et al. (2022)**	China	Emerging	80	High	Corporate	Private	311	0.26
4	**Cozzo Cabrera et al. (2025)**	Uruguay	Emerging	61	High	High-stress	Public	320	0.45
5	**Farid et al. (2022)**	Pakistan	Emerging	55	High	Corporate	Private	250	0.42
6	**Khattak et al. (2022)**	Pakistan	Emerging	55	High	Corporate	Private	343	0.5
7	**Koon & Ho (2021)**	Malaysia	Emerging	104	High	Corporate	Private	150	0.75
8	**Lisbona et al. (2021)**	Spain	Developed	57	High	Mixed	Mixed	310	0.51
9	**Lopez-Zafra et al. (2022)**	Spain	Developed	57	High	Mixed	Mixed	215	0.34
10	**Lux et al. (2023)**	Australia	Developed	36	Low	Corporate	Private	281	0.38
11	**Macamo & Klasmeier (2024)**	Germany	Developed	35	Low	Mixed	Mixed	161	0.31
12	**Niswaty et al. (2021)**	Indonesia	Emerging	78	High	Corporate	Public	192	0.39
13	**Pulido-Martos et al. (2023)**	Spain	Developed	57	High	Mixed	Mixed	968	0.3
14	**Shao et al. (2025)**	China	Emerging	80	High	Mixed	Public	1043	0.3
15	**Sigaeva et al. (2022)**	Russia	Emerging	93	High	High-stress	Private	393	0.28
16	**Silva et al. (2023)**	Portugal	Developed	63	High	Corporate	Private	292	0.48
17	**Towsen et al. (2020)**	South Africa	Emerging	49	Low	High-stress	Private	236	0.49
18	**Vermeulen & Scheepers (2020)**	South Africa	Emerging	49	Low	Corporate	Private	162	0.51
19	**Xue et al. (2023)**	China	Emerging	80	High	High-stress	Public	671	0.34

Characteristics of 19 studies and subgroup analysis (cultural power, industry type, sector and country classification)

Industry type (H2b): Studies were categorized into three groups based on the primary occupational demands reported in each study, following the theoretical rationale outlined in Section 2.4.2. High-stress industries included healthcare, hospitality, education, mining, and military; corporate industries included manufacturing, telecommunications, software, and information technology; mixed industries comprised studies sampling across multiple industry types. This classification yielded 6 high-stress, 8 corporate, and 5 mixed-industry studies. Organizational sector (H2c): Studies were classified as public, private, or mixed based on the organizational sector of their sample. This yielded 6 public, 9 private, and 4 mixed-sector studies. Country classification (H2d): Studies were categorized as emerging markets or developed countries based on established economic development indices ( Divergences, 2023). This yielded 13 studies from emerging markets and 6 from developed countries.

Each subgroup contained at least 4 studies, meeting the minimum recommended threshold for categorical subgroup analysis ( Fu et al., 2011). However, due to the relatively small number of included studies and unbalanced subgroup sizes, the statistical power to detect moderating effects is limited. Simulation studies suggest that meta-analytic subgroup tests with fewer than 10 studies per group have low power to detect even moderate differences in effect sizes ( Vembye et al., 2023). Accordingly, non-significant moderation results in this study should be interpreted as inconclusive rather than as evidence that moderation does not exist.

Given the relatively small number of studies ( *k* = 19), the Knapp and Hartung adjustment was applied to all moderation analyses to obtain more conservative and reliable standard error estimates, thereby reducing the risk of Type I error ( Knapp & Hartung, 2003). Accordingly, moderation effects were evaluated using F-statistics ( *F _M_*) rather than the conventional *Q _M_* test. Effect size meta-regression coefficients were back-transformed from Fisher’s *z* scale to *r* for reporting purposes. Selected articles and subgroup categories are uploaded in Table 2. (also available in Kristanto, S. D. (2026b) using Zenodo).

### 3.7 Narrative synthesis

In addition to the quantitative meta-analysis, which focused on the direct bivariate relationship between authentic leadership and work engagement, a narrative synthesis examined the mediating mechanisms and organizational outcomes identified across the included studies, thereby addressing RQ4. Data relevant to mediating mechanisms and downstream organizational outcomes were extracted from each study during full-text assessment. For each study reporting a mediated model, the following information was recorded: the mediating variable(s), the theoretical framework invoked to justify the mediation pathway (JD-R model, SET, or both), the type of mediation reported (full, partial, or serial), and the country and industry context. Similarly, for studies examining outcomes beyond work engagement, the specific outcome variable, its operationalization, and the role of work engagement in the pathway were recorded.

The extracted mechanisms were organized into thematic categories through an inductive process in which mediators were grouped based on their conceptual level of analysis: personal psychological resources, wellbeing constructs, relational and trust mechanisms, and organizational climate and contextual factors. Downstream outcomes were similarly categorized as organizational performance, work behavior, or leadership development outcomes. Evidence strength for each mechanism was assessed based on the number of independent replications: mechanisms examined in two or more independent studies were classified as having moderate evidence, while single-study mechanisms were classified as having preliminary evidence requiring replication.

## 4. Results

The quality assessment results for all 19 included studies using the MMAT 2018 are available in Kristanto, S. D. (2026c). All studies passed the initial MMAT screening stage, confirming their foundational methodological integrity and suitability for further appraisal. Of the 19 studies, 14 (73.68%) were rated as high methodological quality and 5 (26.32%) as moderate quality; no study received a low-quality rating. The 18 purely quantitative studies were appraised using the MMAT Quantitative Non-Randomized category (Section 3), which evaluates sampling representativeness (criterion 3.1), measurement validity (criterion 3.2), data completeness (criterion 3.3), control for confounding variables (criterion 3.4), and appropriate exposure measurement (criterion 3.5). One mixed-methods study ( Koon & Ho, 2021) was further evaluated using the Mixed Methods category (Section 5) to assess the rationale for integration and the coherence between qualitative and quantitative components. The majority of studies employed validated measurement instruments for both authentic leadership (e.g., the Authentic Leadership Questionnaire) and work engagement (e.g., the Utrecht Work Engagement Scale), reported complete outcome data, and applied appropriate statistical analyses to test their hypothesized models. Overall, the quality assessment supports the methodological integrity of the evidence base synthesized in this meta-analysis.

### 4.1 Heterogeneity analysis

The heterogeneity analysis across the 19 studies revealed significant between-study variability. Cochran’s Q-test was significant, *Q*(18) = 109.87, *p* < .001, and the *I ^2^* statistic was 87.20%, classified as high heterogeneity. The between-study standard deviation was τ = 0.14 (95% *CI* [0.10, 0.23]). These results indicate that approximately 87.20% of the observed variance in effect sizes reflects true differences attributable to contextual factors, such as cultural, industrial, or methodological conditions, rather than sampling error alone. Accordingly, the use of a random-effects model with the REML estimator was confirmed as appropriate for this meta-analysis.

### 4.2 Main effect analysis

The meta-analysis confirmed a significant and positive relationship between authentic leadership and work engagement, thereby supporting Hypothesis 1. The pooled correlation of *r* = 0.41 (95% *CI* [0.35, 0.47]) falls in the medium range according to Cohen’s conventional benchmarks ( Cohen, 2013; Xiao, 2024). Furthermore, the estimate is comparable to, and slightly larger than, the pre-pandemic pooled correlation of r = 0.39 reported by Decuypere and Schaufeli (2021).

**Figure 3.**
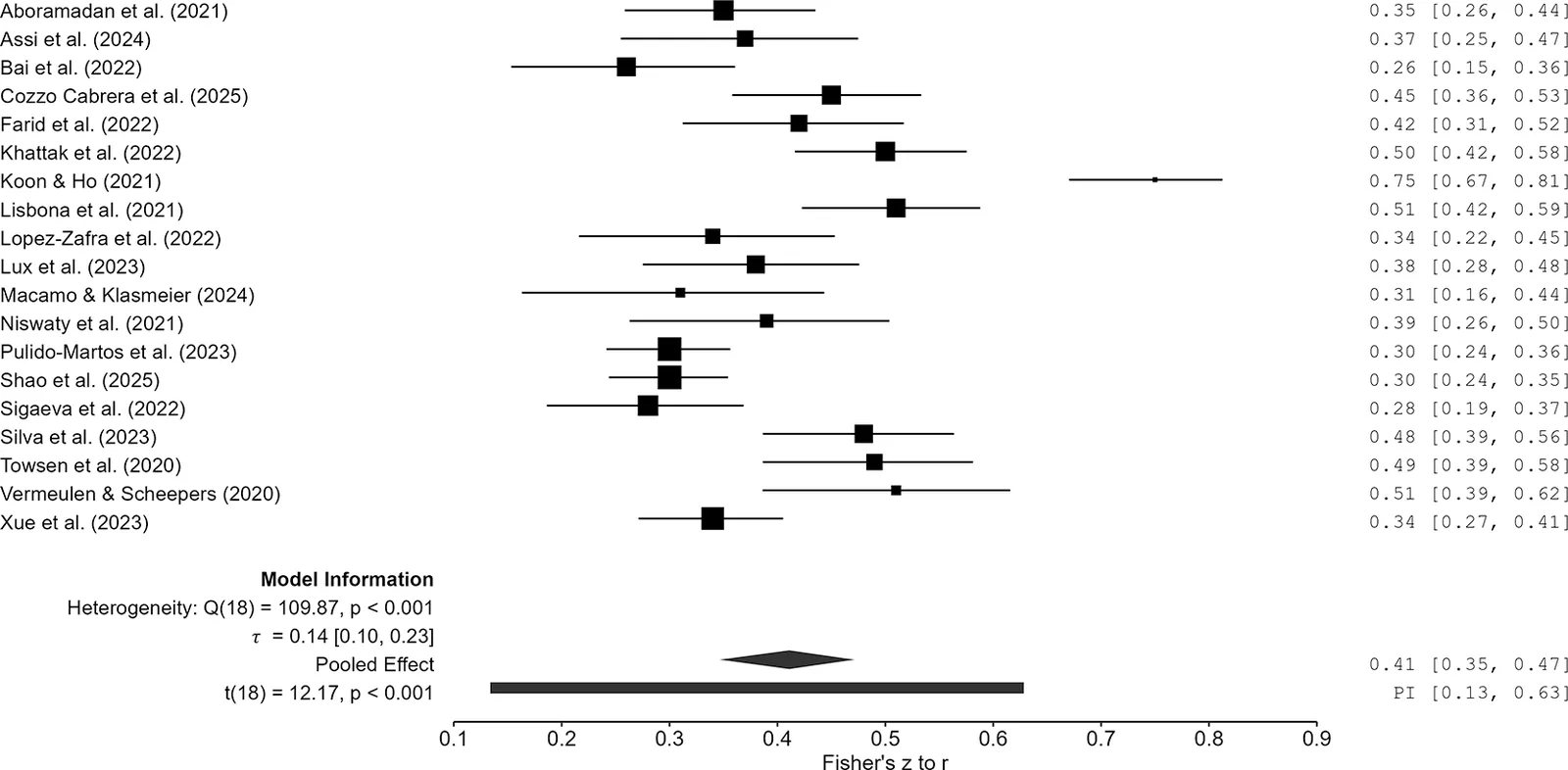
Forest Plot. Forest Plot of this meta-analysis study. The pooled effect size, r = 0.41 (95% CI [0.35, 0.47]), indicates a medium-to-strong positive correlation.

Given that the 95% confidence interval does not cross zero, this relationship is statistically significant. The pooled effect test yielded *t*(18) = 12.17, *p* < .001, providing robust evidence that authentic leadership is positively associated with work engagement across post-pandemic organizational contexts. The 95% Prediction Interval ranged from 0.13 to 0.63, as depicted in the Forest Plot ( Figure 3). Importantly, the lower bound of the prediction interval remains positive, indicating that the authentic leadership–work engagement relationship is expected to be positive even in future studies conducted under differing organizational conditions. Individual study effect sizes ranged from *r* = 0.26 ( Bai et al., 2022) to *r* = 0.75 ( Koon & Ho, 2021), with all 19 studies reporting positive correlations.

### 4.3 Publication bias analysis

**Figure 4.**
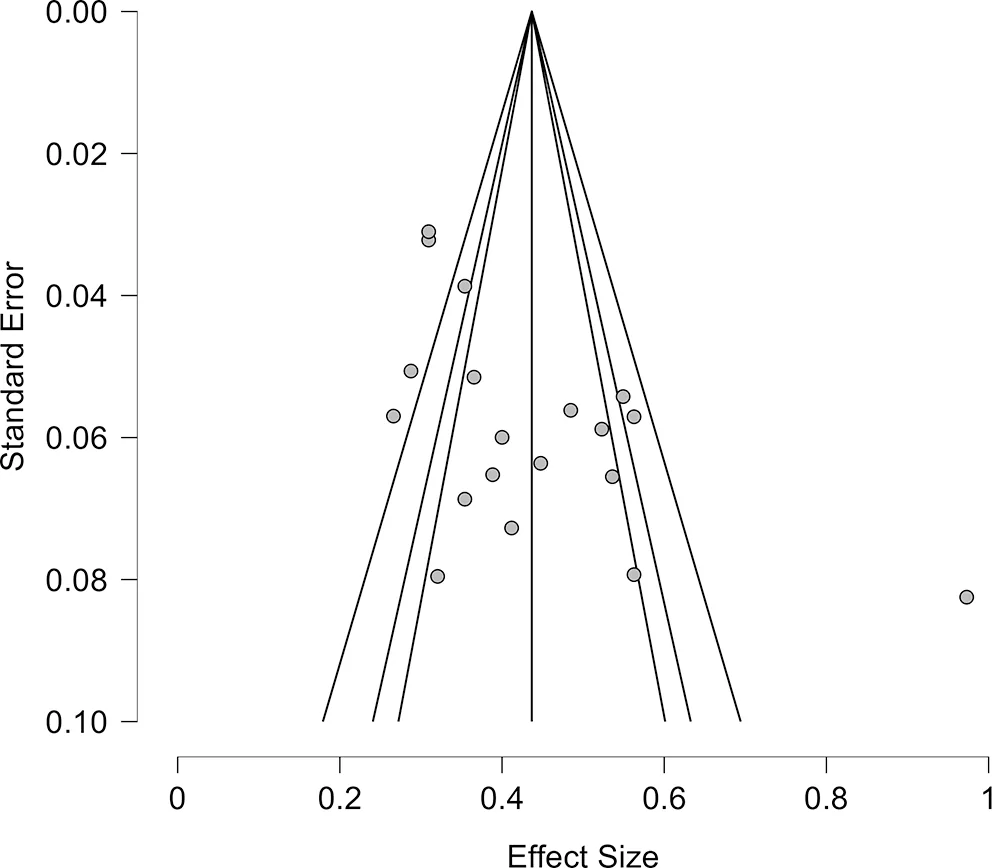
Funnel Plot. The Funnel Plot shows moderate asymmetry, with most studies falling to the right of the pooled estimate.

Publication bias was assessed using multiple complementary approaches. Visual inspection of the Funnel Plot ( Figure 4) revealed moderate asymmetry, with a concentration of studies on the right side (positive effect) of the pooled estimate. This asymmetry was confirmed statistically by the meta-regression asymmetry test ( *z* = 2.63, *p* = .009) and Egger’s weighted regression test ( *t* = 3.09, *df* = 17, *p* = .007), suggesting the potential presence of small-study effects or publication bias. However, the interpretation of this asymmetry requires caution, as Egger’s test cannot definitively distinguish between asymmetry arising from publication bias and asymmetry attributable to genuine between-study heterogeneity ( Sterne et al., 2011). Given the substantial heterogeneity observed in this meta-analysis ( *I ^2^* = 87.20%), it is plausible that the asymmetry reflects contextual variation in true effect sizes rather than selective reporting. Nonetheless, the possibility of some degree of publication bias cannot be entirely ruled out. Rosenthal’s Fail-Safe N indicated that 7,783 additional null studies would be required to reduce the pooled effect to non-significance, a value far exceeding the recommended threshold of 105 (5 *k* + 10). This result provides strong evidence that the overall finding is highly robust and unlikely to be an artifact of publication bias.

### 4.4 Sensitivity analysis

**Figure 5.**
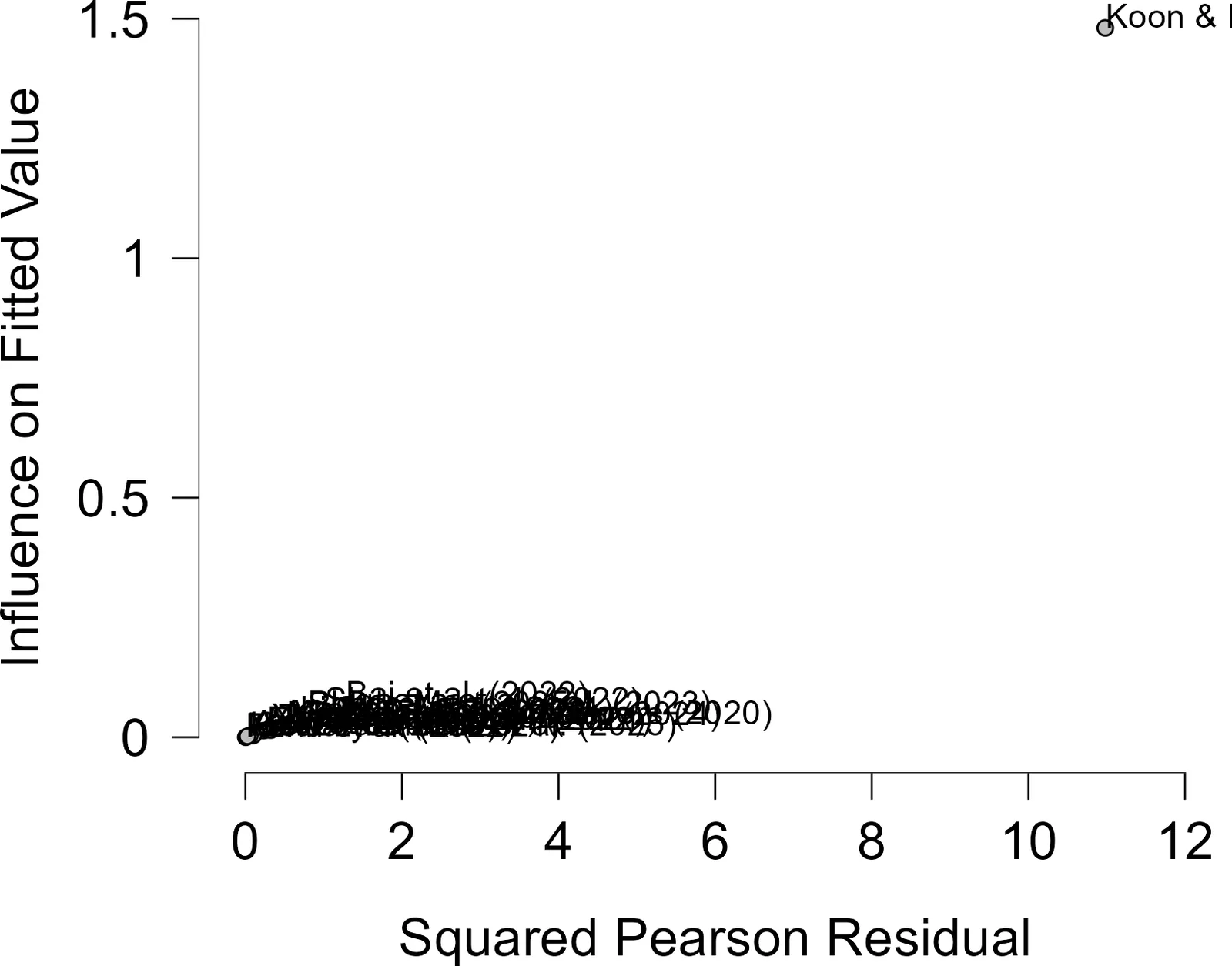
Baujat Plot. The Baujat plot shows that 18 of the 19 studies are near the origin, and the study by Koon and Ho (2021) was identified as an outlier.

A diagnostic Baujat Plot ( Figure 5) was generated to identify individual study contributions to overall heterogeneity and their influence on the pooled effect size. The plot revealed that 18 of the 19 studies (94.74%) clustered near the origin, indicating low heterogeneity contribution and minimal influence on the pooled estimate. The study by Koon and Ho (2021) was identified as an outlier, contributing disproportionately to both overall heterogeneity and influence on the pooled effect size.

To assess the impact of this outlier, the meta-analysis was re-executed excluding Koon and Ho (2021). The adjusted pooled effect based on *k* = 18 studies remained statistically significant and positive: *r* = 0.39, 95% *CI* [0.34, 0.43], t(17) = 17.09, *p* < .001. Heterogeneity decreased from *I ^2^* = 87.20% to *I ^2^* = 72.08%, though it remained substantial, *Q*(17) = 60.15, *p* < .001. The 95% prediction interval narrowed to [0.22, 0.53], with a higher lower bound reflecting greater cross-contextual stability. Notably, the meta-regression asymmetry test became non-significant ( *z* = 1.93, *p* = .054), suggesting that the funnel plot asymmetry observed in the primary analysis was largely attributable to this single influential study rather than systematic publication bias. Egger’s weighted regression test remained marginally significant ( *t* = 2.68, *df* = 16, *p* = .016), indicating some residual asymmetry. Rosenthal’s Fail-Safe N remained exceptionally large ( *N* = 6,569), far exceeding the recommended tolerance level of 100.

These results affirm that the positive association between authentic leadership and work engagement is robust and not contingent upon any single influential study. Accordingly, the primary meta-analysis was retained based on the full sample of *k* = 19 studies, with the sensitivity analysis confirming the stability of the primary findings.

### 4.5 Moderation analysis

Categorical subgroup analyses were conducted to test the moderation hypotheses (H2a–H2d). All analyses applied the Knapp and Hartung adjustment to obtain more conservative estimates. Meta-regression coefficients and confidence intervals were back-transformed from the Fisher’s *z* scale to *r* for reporting purposes. Results for each moderator are reported below.

**4.5.1 Cultural power distance (H2a)**

Hypothesis 2a predicted that the strength of the authentic leadership–work engagement correlation would vary significantly across high- and low-power-distance cultural contexts. The Omnibus Test of Moderators revealed that cultural power distance did not significantly explain heterogeneity in effect sizes, *F _M_* (1, 17) = 0.06, *p* = .81. The intercept, representing the high-power distance reference group, showed a significant effect size of *r* = 0.41 ( *p* < .001; 95% *CI* [0.33, 0.48]). The estimated pooled effect for low power distance cultures was *r* = 0.43, yielding a non-significant difference of 0.02 (95% *CI* [−0.15, 0.16], *p* = .81). Substantial residual heterogeneity remained after including this moderator, *Q _e_* (17) = 106.84, *p* < .001, *I ^2^* = 88.07%, confirming that cultural power distance does not account for the observed between-study variability. Hypothesis 2a is not supported.

**4.5.2 Industry type (H2b)**

Hypothesis 2b predicted that the relationship between authentic leadership and work engagement would differ significantly across different industry types (e.g., high-stress, corporate, or mixed). The Omnibus Test of Moderation results revealed that industry type did not moderate the relationship, *F _M_* (2, 16) = 1.56, *p* = .24, indicating that industry type did not significantly explain between-study variance in effect sizes. The Intercept (representing corporate type) revealed a large and very robust effect size of *r* = 0.47 ( *p* < .001, 95% *CI* [0.37, 0.55]). High-stress industries had estimated pooled effect of r = 0.38, so the difference was −0.09 (95% *CI* [−0.25, 0.05], *p* = .20). The mixed industry estimate pooled effect *r* = 0.35, so the difference was −0.12 (95% *CI* [−0.28, 0.03], *p* = .13). Residual heterogeneity remained substantial, *Q _e_* (16) = 86.02, *p* < .001, *I ^2^* = 85.75%, confirming that industry type does not meaningfully account for the observed between-study variability. Thus, H2b is not supported.

**4.5.3 Organizational sector (H2c)**

Hypothesis 2c predicted that organizational sector (public, private, or mixed) would moderate the relationship. The Omnibus Test of Moderators revealed no significant moderation effect, *F _M_* (2, 16) = 1.24, *p* = .32. The intercept, representing the mixed sector, showed a significant effect size of *r* = 0.37 ( *p* < .001; 95% *CI* [0.22, 0.50]). The private sector yielded an estimated pooled effect of *r* = 0.46, with a non-significant difference of 0.09 (95% CI [−0.08, 0.23]; *p* = .26), while the public sector yielded an estimated pooled effect of *r* = 0.37, with a negligible and non-significant difference of −0.00 (95% *CI* [−0.20, 0.16]; *p* = .97). Notably, the public and mixed-sector effect sizes were nearly identical, suggesting comparable relationship strengths across these organizational contexts. Residual heterogeneity remained substantial, *Q _e_* (16) = 93.32, *p* < .001, *I ^2^* = 86.15%. Hypothesis 2c is not supported.

**4.5.4 Country classification (H2d)**

Hypothesis 2d predicted that the relationship would differ between emerging markets and developed countries. The Omnibus Test of Moderators showed no significant moderation, *F _M_* (1, 17) = 0.21, *p* = .65. The intercept, representing developed countries, showed a significant effect size of *r* = 0.39 ( *p* < .001; 95% *CI* [0.27, 0.50]). The estimated pooled effect for emerging markets was *r* = 0.42, with a non-significant difference of 0.03 (95% *CI* [−0.12, 0.16]; *p* = .65). Residual heterogeneity remained unchanged, *Q _e_* (17) = 109.64, *p* < .001, *I ^2^* = 87.56%. Hypothesis 2d is not supported.

**4.5.5 Summary of moderation analyses**

None of the four tested moderators, cultural power distance, industry type, organizational sector, or country classification, significantly explained the between-study variance in the authentic leadership–work engagement relationship. Despite the inclusion of these moderators, residual heterogeneity remained substantial ( *I ^2^* > 85% across all models), indicating that the observed between-study variability is driven by factors not captured in the current moderation framework. While these results are consistent with the possibility that authentic leadership functions as a broadly relevant antecedent of work engagement, the limited number of studies per subgroup (particularly *k* = 4 for low power distance) constrains statistical power, and the null findings should be interpreted with caution rather than as confirmation of contextual invariance.

### 4.6 Narrative synthesis of mediating mechanisms

The integrated research model ( Figure 1) identifies several mediating pathways through which authentic leadership facilitates work engagement. Of the 19 included studies, 15 studies (78.95%) examined mediated models, indicating that authentic leadership predominantly operates through intermediary psychological and relational mechanisms rather than through a simple direct effect. Narrative synthesis organized into four thematic categories: personal psychological resources, wellbeing constructs, relational and trust mechanisms, and organizational climate and contextual factors.

**4.6.1 Personal psychological resources**

Two personal psychological resources were identified as mediators of the relationship between authentic leadership and work engagement: psychological capital and psychological empowerment. Psychological capital (PsyCap), comprising hope, efficacy, resilience, and optimism, emerged as the most frequently examined mediator in the evidence base, tested in three independent studies across Indonesia ( Niswaty et al., 2021), Russia ( Sigaeva et al., 2022), and China ( Xue et al., 2023). Xue et al. (2023) found that PsyCap fully mediated the relationship between a healthy work environment, of which authentic leadership is a core dimension, and work engagement among 671 Chinese healthcare workers. Sigaeva et al. (2022) reported partial mediation in their study of Generation Z employees in Russia’s hospitality sector, while Niswaty et al. (2021) similarly found partial mediation in Indonesia’s public service context. The replication of PsyCap as a mediator across three emerging economies with high power distance cultures provides the strongest evidence among the tested mediators that authentic leaders enhance engagement by building followers’ personal psychological resources, consistent with the motivational pathway of the JD-R model.

Psychological empowerment, comprising meaning, competence, self-determination, and impact ( Spreitzer, 1995), was examined in two studies. Towsen et al. (2020) demonstrated that authentic leaders in South African coal mining, through self-awareness and balanced processing, enable employees to perceive their roles as significant, thereby increasing vigor and dedication. Khattak et al. (2022) found that psychological empowerment mediates the relationship between authentic leadership and project success in Pakistan’s software industry, suggesting that authentic leaders create environments fostering both psychological safety and the autonomy required for empowerment. Both studies reported partial mediation, indicating that empowerment captures an important but not exhaustive portion of the authentic leadership and engagement pathway.

**4.6.2 Wellbeing constructs**

Three wellbeing-related constructs were identified as mediating mechanisms, each examined in a single study. Farid et al. (2022) reported a serial mediation pathway through hedonic wellbeing (pleasure, job satisfaction) and eudaimonic wellbeing (personal meaning, self-purpose) in Pakistan’s telecommunications industry. This finding suggests that authentic leadership enhances engagement through a sequential process in which leaders first promote pleasure-based satisfaction, which in turn activates deeper meaning-based motivation. While this serial pathway is theoretically novel in integrating the hedonic and eudaimonic traditions, it requires replication to establish its robustness.

Koon and Ho (2021) found that workplace wellbeing partially mediated the relationship between authentic leadership and engagement in Malaysian service SMEs, proposing that authentic leaders create wellbeing through just and fair practices. However, this finding should be interpreted cautiously given that this study was identified as an outlier in the meta-analysis ( *r* = 0.75) with the smallest sample size ( *N* = 150) in the evidence base.

Lopez-Zafra et al. (2022) demonstrated that vigor at work fully mediated the effect of authentic leadership on engagement among Spanish employees. While this finding supports the JD-R model’s emphasis on energetic resources, it warrants careful theoretical scrutiny because vigor is also conceptualized as a core dimension of work engagement itself ( Schaufeli et al., 2002) which raises questions about the mediator’s conceptual independence from the outcome.

**4.6.3 Relational and trust mechanisms**

Two relational mechanisms were identified, both grounded in Social Exchange Theory and each examined in a single study. Lux et al. (2023) identified the most complex mediation model in the evidence base: a three-stage serial pathway from personal identification through affect-based trust to affective organizational commitment, which fully mediated the authentic leadership–engagement relationship in an Australian sample across various industries. This sequential model suggests that authentic leaders first activate follower identification, which deepens into affective trust, which consolidates into organizational commitment before manifesting as engagement. While theoretically rich, the model’s complexity necessitates replication to establish generalizability.

Vermeulen and Scheepers (2020) found that perceived organizational support (POS) partially mediated the relationship in South Africa’s IT sector. This mechanism operates at the organizational level of analysis, distinguishing it from the individual-level psychological resources identified in other studies. The finding suggests that authentic leadership enhances engagement partly by shaping employees’ perceptions that their organization values their contributions — a reciprocity signal consistent with SET.

**4.6.4 Organizational climate and contextual factors**

Three contextual or collective-level mechanisms were identified, each in a single study. Shao et al. (2025), in the largest study in the evidence base ( *N* = 1,043), identified school climate and teacher efficacy as serial mediators between authentic leadership and teacher engagement in Chinese primary and secondary schools. This pathway suggests that authentic principals first shape a supportive school climate, which, in turn, enhances teachers’ belief in their professional capabilities, ultimately increasing engagement. While the large sample strengthens the internal validity of this finding, these mediators are specific to the education sector and may not generalize to other organizational contexts.

Lisbona et al. (2021) demonstrated that team initiative, collective proactive behavior fostered through participative decision-making and psychological security, fully mediated the relationship between authentic leadership and engagement in Spanish organizations. This is the only team-level mediator in the evidence base, offering a distinct perspective on how authentic leadership operates through collective rather than individual psychological processes. ( Silva et al., 2023) found that meaningful work and work–family enrichment served as individual and serial mediators in Portuguese service organizations. This study uniquely extends the mediation framework to the work–family interface, suggesting that authentic leaders promote engagement partly by enabling positive spillover from work to family life.

**4.6.5 Summary of mediating mechanisms**

In sum, the mediating evidence reveals that authentic leadership influences work engagement through multiple channels spanning personal resources, wellbeing, relational trust, and organizational climate. This diversity of pathways is consistent with the proposition that authentic leadership functions simultaneously as a job resource (JD-R model) and a trigger for social exchange (SET). However, the evidence base for individual mechanisms remains thin: only psychological capital ( *k* = 3) and psychological empowerment ( *k* = 2) have been replicated across independent samples. The remaining eight mechanisms represent single-study findings that, while theoretically informative, require independent replication. Future research should prioritize testing competing mediation models within the same study to evaluate the relative explanatory power of different mechanisms, and employ longitudinal designs to establish temporal precedence among authentic leadership, mediating processes, and work engagement.

### 4.7 Narrative synthesis of organization outcomes

The integrative framework posits that the authentic leadership and work engagement linkage extends beyond psychological states to tangible organizational outcomes. Four studies examined downstream outcomes and identified three categories: organizational performance, work behavior, and leadership development. The synthesis below follows the same categorical structure.

Two studies linked the authentic leadership and work engagement pathway to organizational performance outcomes in different sectoral contexts. Aboramadan et al. (2021) demonstrated that in Jordanian public hospitals, authentic leadership significantly predicted hospital performance, including quality of care and operational efficiency, with work engagement serving as a mediating pathway. This finding suggests that authentic leaders contribute to healthcare quality not merely by motivating individual staff, but by fostering the engaged workforce that drives institutional performance. ( Khattak et al., 2022) found a parallel pattern in Pakistan’s software industry, where authentic leadership was associated with project success, encompassing on-time delivery, quality, and stakeholder satisfaction, through the mediating roles of psychological empowerment and work engagement. Notably, this study reported the second-highest effect size in the evidence base ( *r* = 0.50), suggesting that the authentic leadership and engagement pathway may be particularly consequential for project-based outcomes where sustained focus and dedication are critical. While these two studies point to a meaningful performance pathway, both examined sector-specific outcomes (hospital performance, software project success) that may not directly generalize across industries.

Second in work behavior, ( Bai et al., 2022) provided evidence from China’s high-tech manufacturing sector that authentic leadership predicts innovative work behavior, encompassing idea generation, promotion, and implementation, through the mediating role of work engagement. This finding suggests that engaged employees are more willing to move beyond formal job descriptions and take creative risks. In an increasingly dynamic post-pandemic environment in which organizations rely on innovation for competitive advantage, this pathway may hold strategic significance. However, this study reported the smallest effect size in the evidence base ( *r* = 0.26), and the finding remains limited to a single manufacturing context.

Last in leadership development, ( Cozzo Cabrera et al., 2025) identified a distinctive and strategically relevant outcome: in the Uruguayan military, authentic leadership influenced personnel’s aspirations to assume future leadership roles, with work engagement mediating this relationship. This finding suggests that authentic leaders not only engage followers in their current tasks but also inspire commitment to future leadership development, supporting organizational succession planning and long-term sustainability. While the military context has institutional features, including formalized rank structures and career progression systems, that may limit generalizability to civilian organizations, the identification of leadership aspirations as a downstream outcome of engagement represents a unique contribution to the literature.

Collectively, these findings indicate that work engagement should not be viewed merely as an isolated employee attitude but as a mechanism that translates authentic leader behaviors into broader organizational outcomes. However, it should be noted that each downstream outcome was examined in only a single study, and no outcome pathway has been independently replicated. The evidence for organizational outcomes downstream of the authentic leadership-work engagement relationship should therefore be regarded as preliminary and indicative, offering promising directions for future research rather than established conclusions.

## 5. Discussion

This meta-analysis synthesized recent empirical evidence on the relationship between authentic leadership and work engagement within the post-pandemic new normal (2020–2025). By employing a rigorous methodological standard that included only bivariate Pearson correlation coefficients, this study ( *k* = 19; *N* = 6,916) provides a precise and contemporary estimate of this critical organizational relationship. The discussion is structured around the four research questions guiding this study.

### 5.1 Main effect: authentic leadership and work engagement (RQ1)

The meta-analysis yielded a significant and positive pooled correlation of *r* = 0.41 ( *p* < .001) between authentic leadership and work engagement, classified as a medium effect ( Cohen, 2013). This finding confirms Hypothesis 1 and provides strong evidence that authentic leadership remains a robust antecedent of work engagement in contemporary organizational contexts. The pooled effect size is slightly stronger than that reported in a prior meta-analysis by Decuypere and Schaufeli (2021), who found *r* = 0.39 based on pre-pandemic data. This marginal increase suggests that the relevance of authentic leadership has not diminished and may have modestly strengthened in the wake of the global disruptions experienced between 2020 and 2022.

The robustness of this finding is supported by multiple indicators. Rosenthal’s Fail-Safe N of 7,783 exceeds the required threshold (5 *k* + 10 = 105) by a factor of approximately 74, providing exceptionally strong evidence that the positive association is not an artifact of selective publication ( Sterne et al., 2011). Furthermore, the 95% Prediction Interval (0.13 to 0.63) indicates that future studies are very likely to find a positive correlation between authentic leadership and work engagement, regardless of context. Although Egger’s weighted regression test indicated statistically significant funnel plot asymmetry ( *t* = 3.09, *df* = 17, *p* = .007), this finding should be interpreted alongside the full suite of robustness checks (see also Section 4.3). The Baujat plot ( Figure 5) identified only Koon and Ho (2021) as a notable outlier in terms of both heterogeneity contribution and influence on the pooled estimate, and the leave-one-out sensitivity analysis confirmed that the pooled effect remained significant and within the medium range after its exclusion. Taken together, these converging lines of evidence suggest that the overall finding is robust, although the possibility of some degree of publication bias cannot be entirely discounted.

The consistency of this finding carries important theoretical implications. Across the 19 samples included in this synthesis, the positive relationship between authentic leadership and work engagement was observed without exception, with individual effect sizes ranging from *r* = 0.26 to *r* = 0.75. This pattern suggests that employees’ responsiveness to leaders who demonstrate openness, fairness, and moral integrity is robust across the range of organizational settings represented in the current evidence base. Authentic leadership appears to provide motivational energy that translates into the vigor, dedication, and absorption that characterize work engagement ( Assi et al., 2024; Silva et al., 2023), although the extent to which this relationship varies across contexts not well represented in the current sample remains an open question.

### 5.2 Contextual moderation (RQ2)

Despite the high heterogeneity observed across the 19 studies ( *I ^2^* = 87.20%), with individual effect sizes ranging from *r* = 0.26 to *r* = 0.75, the categorical subgroup analyses revealed that none of the four tested moderators significantly explained the between-study variance. This finding, while contrary to the hypothesized expectations (H2a–H2d), carries substantial theoretical significance.

The non-significant result for cultural power distance (H2a; *F _M_* (1, 17) = 0.06, *p* = .81) indicates that, within the current dataset, cultural power distance did not significantly account for between-study variation in the authentic leadership–work engagement relationship. The estimated pooled effects for high-power distance ( *r* = 0.41) and low-power distance ( *r* = 0.43) cultures were closely comparable. However, this finding should be interpreted cautiously: only four studies represented low-power distance cultures, substantially limiting statistical power to detect a moderation effect. Within these constraints, the results are consistent with the possibility that authentic leadership promotes engagement across different cultural contexts, though the degree to which the underlying mechanisms are culturally equivalent or operate through distinct pathways (e.g., reciprocity-based in high-PDI contexts vs. autonomy-based in low-PDI contexts) cannot be determined from the present data and warrants investigation in future research.

The non-significant moderation by industry type (H2b; *F _M_* (2, 16) = 1.56, *p* = .24) suggests that, within the current evidence base, industry classification did not significantly account for differences in the authentic leadership–work engagement relationship. Although estimated pooled effects were directionally higher in corporate settings ( *r* = 0.47) than in high-stress ( *r* = 0.38) or mixed ( *r* = 0.35) industries, these differences did not reach statistical significance. This pattern is broadly consistent with the JD-R model’s proposition that authentic leadership functions as a fundamental job resource ( Lopez-Zafra et al., 2022), although the observed directional trend warrants further investigation with larger samples to determine whether industry-specific demands may attenuate the leadership–engagement association.

Similarly, neither organizational sector (H2c; *F _M_* (2, 16) = 1.24, *p* = .32) nor country economic classification (H2d; *F _M_* (1, 17) = 0.21, *p* = .65) significantly moderated the relationship. The positive association between authentic leadership and work engagement was observed across public, private, and mixed-sector organizations, and across both emerging markets and developed countries represented in the dataset. While these findings are consistent with the interpretation that authentic leadership may function as a broadly relevant organizational resource, the modest number of studies within each subgroup limits the statistical power to detect potential moderation effects.

While these null moderation results should not be interpreted as definitive evidence of contextual invariance, the consistent pattern of non-significant moderation across four distinct macro-level variables carries noteworthy implications. Taken together, the findings suggest that the positive relationship between authentic leadership and work engagement is not easily attributable to any single contextual boundary condition tested in this study. This pattern is consistent with the broader proposition that the core elements of authenticity, transparency, moral integrity, balanced processing, and self-awareness may hold relevance across diverse organizational settings. However, given the limited statistical power of the moderation tests and the substantial unexplained heterogeneity that persists across all models, future research with larger and more balanced samples is needed before stronger claims of cross-contextual generalizability can be substantiated. Importantly, the finding that authentic leadership is positively associated with work engagement in emerging economies, contexts historically underrepresented in the leadership literature, extends the applicability of authentic leadership theory beyond its predominantly Western empirical origins.

### 5.3 Theoretical interpretation (RQ3)

The findings are consistent with the integrative application of the JD-R model and SET in explaining the relationship between authentic leadership and work engagement. While the meta-analysis tests the bivariate association rather than the structural pathways posited by each theory, the pattern of results, including the strength and consistency of the pooled effect across diverse contexts, aligns with the predictions of both theoretical frameworks, as elaborated below.

From the perspective of the JD-R model, the results confirm that authentic leadership behaviors: transparency, balanced processing, and ethical integrity, function as critical job resources that shape employee commitment and enthusiasm at work ( Schaufeli et al., 2002; Silva et al., 2023). Authentic leaders reduce uncertainty and role ambiguity while simultaneously fostering conditions for intrinsic motivation, thereby activating the motivational pathway from resources to engagement. The non-significant moderation by industry type is consistent with this interpretation, suggesting that across the range of industries represented in the current sample, authentic leadership may provide a resource base that supports engagement irrespective of specific job demands, although this conclusion remains tentative given the limited number of studies per industry category.

From the perspective of SET, the findings are consistent with the proposition that authentic leadership triggers a reciprocity cycle in which employees respond to perceived fairness, honesty, and moral integrity with heightened vigor and absorption ( Towsen et al., 2020). Aligning with SET, authentic leaders foster high-quality social exchanges that encourage employees to feel obligated to reciprocate with innovative behaviors beyond standard expectations. This reciprocal dynamic becomes especially powerful when engagement converts a leader’s supportive resources into the innovative energy needed to address complex organizational challenges. The non-significant moderation by cultural context is consistent with the possibility that this reciprocity mechanism operates across different cultural settings, although the small number of low power distance studies ( *k* = 4) limits the strength of this inference. The directional similarity of effects across high and low-power distance contexts tentatively suggests that employees may respond positively to authentic leadership behavior irrespective of whether such transparency is normatively expected or culturally distinctive.

Together, the JD-R model explains how authentic leadership activates engagement through resource provision, while SET explains why followers respond to such leadership with enhanced engagement through relational reciprocity. As projects become more decentralized and require autonomous team performance, leaders’ ability to cultivate a committed workforce through both resource provision and relational trust becomes increasingly vital for ensuring organizational success and resilience.

### 5.4 Mediating mechanisms and organizational outcomes (RQ4)

The narrative synthesis identified 10 distinct mediating mechanisms across four thematic categories and four downstream organizational outcomes. Three features of the mediating evidence warrant discussion. First, the evidence base for individual mechanisms is notably thin. Only psychological capital ( *k* = 3) and psychological empowerment ( *k* = 2) have been examined in more than one independent study; the remaining eight mediators have been examined in only a single study. While this breadth of pathways is consistent with the proposition that authentic leadership functions simultaneously as a job resource and a trigger for social exchange, it also means that most proposed mechanisms have not yet been subjected to the replication that scientific confidence requires. The field has generated many candidate mechanisms but has not yet converged on which pathways are most robust.

Second, the diversity of mediating mechanisms, spanning personal psychological resources, wellbeing states, relational trust processes, and organizational climate factors, suggests that authentic leadership may operate through multiple channels simultaneously rather than through a single dominant pathway. This multi-pathway interpretation aligns with the JD-R model’s proposition that job resources enhance engagement through both motivational processes ( Khattak et al., 2022; Towsen et al., 2020), and with SET’s emphasis on relational reciprocity ( Lux et al., 2023). Future research should test competing mediation models within the same study to evaluate the relative explanatory power of these channels.

Third, the downstream organizational outcomes identified are hospital performance ( Aboramadan et al., 2021), software project success ( Khattak et al., 2022), innovative work behavior ( Bai et al., 2022), and leadership aspirations ( Cozzo Cabrera et al., 2025), suggest that work engagement should not be viewed as a terminal outcome but as a mechanism that translates authentic leader behaviors into tangible organizational performance and development outcomes. However, each outcome was examined in only a single study, and no outcome pathway has been independently replicated. These findings should therefore be regarded as promising directions for future research rather than established causal chains.

Overall, the mediating evidence supports the study’s integrated theoretical framework (RQ3) by demonstrating that the relationship between authentic leadership and work engagement is not a simple direct effect but operates through identifiable intermediary processes. The challenge for the next generation of research is to move from pathway identification to pathway comparison and replication.

### 5.5 Quality assessment considerations

All 19 included studies were classified as high (73.68%) or moderate (26.32%) methodological quality according to the MMAT 2018 criteria, thereby ensuring a robust evidence base for this synthesis. The quality assessment highlighted that the majority of studies employed validated measurement instruments, reported complete outcome data, and applied appropriate statistical analyses. However, several methodological limitations were identified: most cross-sectional studies are at risk of common method bias because they rely on single-source, self-reported survey data collected at a single point in time. Recent post-pandemic studies have increasingly adopted time-lagged configurations and multi-source data collection approaches ( Lisbona et al., 2021; Sigaeva et al., 2022), thereby elevating the overall internal validity of the synthesized findings.

### 5.6 Contributions

This meta-analytic study makes contributions in three theoretical areas. First, by strictly excluding regression-based effect sizes ( *β* coefficients) from the analysis, this study establishes a methodologically rigorous baseline for estimating the zero-order bivariate relationship between authentic leadership and work engagement. This addresses a limitation in prior syntheses that pooled heterogeneous effect size metrics ( Paul & Barari, 2022). Second, by incorporating studies conducted across emerging economies in Asia, Eastern Europe, South America, and Africa, this study broadens the empirical base of authentic leadership research to cultural contexts that have historically been underrepresented in the leadership literature. The observation that the authentic leadership–work engagement relationship was consistently positive across all 12 countries and that none of the tested macro-contextual moderators significantly explained between-study variance offers preliminary support for the construct’s cross-cultural relevance, while acknowledging that the limited number of studies per subgroup precludes definitive conclusions. Third, the findings are consistent with the complementary nature of authentic leadership as both a relational antecedent (through SET) and a motivational resource (through the JD-R model), suggesting that these theoretical perspectives jointly help interpret the leadership–engagement dynamic.

The practical implications are equally noteworthy. Organizations should consider incorporating validated authenticity measures into leadership selection processes to identify candidates who demonstrate self-awareness and moral integrity. Leadership development programs should extend beyond technical skill-building to include inner work that cultivates the courage for transparent communication and the discipline for objective decision-making. In the current talent-scarce environment, particularly in the post-pandemic era, developing authentic leaders is a strategic mechanism for talent retention, as it creates the supportive environment that high-performing employees increasingly demand.

### 5.7 Limitations and future research

Although the findings are robust, several limitations are acknowledged. First, while the total participant count ( *N* = 6,916) is substantial, the number of included studies ( *k* = 19) is relatively small. This necessitated the use of the conservative Knapp and Hartung adjustment to mitigate the risk of Type I error. The small *k* also resulted in unbalanced subgroup sizes in moderation analyses, most notably in the cultural power distance analysis (15 high vs. 4 low), which may have limited statistical power to detect moderation effects. Furthermore, the PDI classification involves borderline cases: South Africa (PDI = 49) falls just 1 point below the threshold of 50, and its reclassification into the high-power distance group would reduce the low-PDI subgroup to only 2 studies (Australia and Germany), further constraining the analysis. Future meta-analyses with larger samples should either use power distance as a continuous moderator in meta-regression or conduct sensitivity analyses with alternative PDI thresholds to assess the robustness of moderation findings.

Second, the search strategy included only open-access articles, potentially excluding relevant studies published in subscription-based journals. This filter was applied to ensure full-text accessibility and replicability but represents a potential source of selection bias that should be acknowledged. Third, the dataset is predominantly focused on emerging economies (e.g., Indonesia, Pakistan, Russia, South Africa). While this addresses a significant gap in the Western-centric literature, the findings may not be fully generalizable to highly individualistic Western organizational cultures that are under-represented in the current sample. Fourth, all included studies employed cross-sectional designs and relied on self-report measures, which inhibit conclusive causal inferences and raise concerns about common method bias. Fifth, it should be noted that two studies with the largest sample size together account for approximately 29% of the total participant count ( Pulido-Martos et al., 2023; Shao et al., 2025). Both studies reported effect sizes ( *r* = 0.30) below the pooled mean, and because random-effects models assign greater weight to larger, more precise studies, these two samples exert disproportionate influence on the pooled estimate. The Baujat plot ( Figure 5) did not identify either study as an outlier in terms of heterogeneity contribution, and the pooled effect remained medium ( *r* = 0.41) despite this weighting pattern, suggesting that the overall finding is not an artifact of sample-size concentration. Nonetheless, future studies with more evenly distributed sample sizes would strengthen the precision of pooled estimates.

Future research should address these study limitations. Longitudinal designs would enable the establishment of causal relationships between authentic leadership and engagement. Given that the tested macro-contextual moderators did not explain the observed heterogeneity, future studies should explore individual-level moderators that may account for between-study variance, such as employee proactive personality, core self-evaluations, or generational differences (e.g., Generation Z vs. Millennials). Additionally, multi-level designs that capture both leader- and team-level dynamics would provide insight into how authentic leadership translates into collective engagement and organizational outcomes.

### 5.8 Conclusion

Drawing on data from 6,916 participants aggregated across 19 samples spanning 12 countries, this meta-analysis provides robust evidence that authentic leadership is significantly and positively associated with work engagement in the post-pandemic era. The medium-to-strong pooled correlation ( *r* = 0.41), together with the absence of significant moderation by the cultural, industrial, sectoral, and economic factors tested, suggests that the core components of authenticity are relevant to employees across the diverse organizational contexts represented in the current evidence base. Authentic leadership extends its benefits beyond the immediate psychological state of the follower: through multiple mediating mechanisms, it provides a chain of desirable organizational outcomes, including innovative work behavior, project success, and leadership development. These findings underscore the strategic case for investing in authentic leadership development to foster engaged, resilient, and innovative workforces. By demonstrating a consistently positive relationship between authentic leadership and work engagement across both emerging markets and developed economies, this study offers preliminary empirical support for authentic leadership development as a pathway to advancing the United Nations 2030 Agenda for Sustainable Development Goal 8: decent work and economic growth.

## Software availability

We use JASP Software (version 0.96.0.0) to do meta-analysis of data. Software available from: https://jasp-stats.org/

Mendeley Reference Manager. Software available from: https://www.mendeley.com/download-reference-manager/windows

## Use of artificial intelligence (AI)

The authors declare that the generative artificial intelligence (AI) tool Gemini 3.0 and Grammarly were used exclusively for language editing and grammatical improvement. The use of AI did not influence the scientific content, study design, data analysis, data interpretation, results, or conclusions of the manuscript. Full responsibility for the content remains with the authors.

## Data Availability

No Data are associated with this article. Zenodo: PRISMA 2020 Flowchart, https://doi.org/10.5281/zenodo.19873636. Kristanto, S. D. (2026a). PRISMA 2020 Flowchart [Data set]. Zenodo. https://doi.org/10.5281/zenodo.19873636 . This project contains the following data:
• PRISMA 2020 Flowchart-180597.docx Data are available under the terms of the Creative Commons Attribution 4.0 International (CC-BY 4.0) and Creative Commons Public Domain Dedication and Certification (CC0 1.0). Zenodo: Meta-Analysis and Subgroup Categories of Selected Studies, https://doi.org/10.5281/zenodo.19913953 . Kristanto, S. D. (2026b). Meta-Analysis and Subgroup Categories of Selected Studies [Data set]. Zenodo. https://doi.org/10.5281/zenodo.19913953 This project contains the following data:
•
Table 2. Meta-Analysis and Subgroup Categories of Selected Studies.xlsx Data are available under the terms of the Creative Commons Attribution 4.0 International (CC-BY 4.0) and Creative Commons Public Domain Dedication and Certification (CC0 1.0). Zenodo: 19 Studies Quality Assessment Results MMAT 2018, https://doi.org/10.5281/zenodo.19607567. Kristanto, S. D. (2026c). 19 Studies Quality Assessment Results MMAT 2018 [Data set]. Zenodo. https://doi.org/10.5281/zenodo.19607567 This project contains the following data:
• 19 Studies Quality Assessment Results MMAT 2018.xlsx Data are available under the terms of the Creative Commons Attribution 4.0 International (CC-BY 4.0) and Creative Commons Public Domain Dedication and Certification (CC0 1.0). Zenodo: JASP Source Code used in Meta Analysis (incl subgroup), https://doi.org/10.5281/zenodo.19513143 . Kristanto, S. D. (2026d). JASP Source Code used in Meta Analysis (incl subgroup) [Code]. Zenodo. https://doi.org/10.5281/zenodo.19513143 This project contains the following data:
• JASP Source Code used in Meta Analysis (incl subgroup).zip, consists of: • MA Main 19 Studies.jasp • MA 19 Studies Sub Group-Country Class.jasp • MA 19 Studies Sub Group-Culture.jasp • MA 19 Studies Sub Group-Industry Type.jasp • MA 18 Studies Exclude Outlier.jasp Data are available under the terms of the Creative Commons Attribution 4.0 International (CC-BY 4.0) and Creative Commons Public Domain Dedication and Certification (CC0 1.0). Zenodo: PRISMA abstracts for ‘Does Authentic Leadership Foster Work Engagement? A Post-Pandemic Systematic Review and Meta-Analysis’. https://doi.org/10.5281/zenodo.19643908. Kristanto, S. D. (2026e). PRISMA_2020_abstract_checklist_Kristanto et al [Data set]. Zenodo. https://doi.org/10.5281/zenodo.19643908 Data are available under the terms of the Creative Commons Attribution 4.0 International (CC-BY 4.0) and Creative Commons Public Domain Dedication and Certification (CC0 1.0). Zenodo: PRISMA checklist for ‘Does Authentic Leadership Foster Work Engagement? A Post-Pandemic Systematic Review and Meta-Analysis’. https://doi.org/10.5281/zenodo.19875812. Kristanto, S. D. (2026f ). PRISMA checklist [Data set]. Zenodo. https://doi.org/10.5281/zenodo.19875812 Data are available under the terms of the Creative Commons Attribution 4.0 International (CC-BY 4.0) and Creative Commons Public Domain Dedication and Certification (CC0 1.0).
