# Supplementary material for: Does Authentic Leadership Foster Work Engagement? A Post-Pandemic Systematic Review and Meta-Analysis
Source: F1000Res. 2026 May 19;15:763. [Version 1] doi: 10.12688/f1000research.180597.1 (PMC13560581; doi:10.12688/f1000research.180597.1)
Supplement: Peer review history

# Reviewer report: Reviewer response for version 1

**DOI:** 10.5256/f1000research.199216.r505790

**Published:** 2026-08-10

**Authors:** Untung Rahardja

**Affiliations:** 1 Universitas Raharja, Tangerang, Indonesia

I enjoyed reading this manuscript. The authors address a relevant question concerning authentic leadership and work engagement, and the focus on studies published between 2020 and 2025 gives the review a clear scope. The research questions are straightforward, and the use of Social Exchange Theory together with the Job Demands-Resources model provides a reasonable framework for discussing the findings.

 The meta-analytic procedure is explained in sufficient detail. I particularly appreciate the decision to focus on bivariate Pearson correlations and to report heterogeneity, prediction intervals, publication-bias tests, and sensitivity analyses. The narrative synthesis is also useful, although there are a few points that could be clarified further.

 I have a few suggestions that may improve the clarity of the manuscript.

 1. The term “post-pandemic” should be used with some caution. The authors state that the 2020–2025 criterion refers to publication year rather than the actual period of data collecttion. Some studies may therefore include data collected before or during the pandemic. It would be helpful to make this distinction more explicit when interpreting the findings.

 2. ⁠The PRISMA flow diagram may benefit from a brief clarification. The manuscript reports that 274 records were screened by title and abstract and that all 274 proceeded to retrieval, while many were later excluded at the full-text stage because they were not relevant. ⁠Please confirm that this flow accurately reflects the screening process.

 3.⁠The level of heterogeneity is relatively high (I² = 87.20%). The authors have appropriately used a random-effects model and performed moderator and sensitivity analyses. However, because considerable residual heterogeneity remains, statements regarding the consistency of the association across different contexts should continue to be made cautiously. 

 4. ⁠The practical implications are useful, although some causal wording could be moderated. The evidence summarized in the review is largely observational and cross-sectional. The findings therefore support a positive association between authentic leadership and work engagement more directly than they support a causal effect.

 These points are mainly suggestions for clarification and do not affect my overall assessment of the manuscript. The research questions are clear, the analytical approach is appropriate, and the main conclusion is supported by the results presented.

Are the rationale for, and objectives of, the Systematic Review clearly stated?

Yes

Is the statistical analysis and its interpretation appropriate?

Yes

If this is a Living Systematic Review, is the ‘living’ method appropriate and is the search schedule clearly defined and justified? (‘Living Systematic Review’ or a variation of this term should be included in the title.)

Not applicable

Are sufficient details of the methods and analysis provided to allow replication by others?

Yes

Are the conclusions drawn adequately supported by the results presented in the review?

Yes

Reviewer Expertise:

Management, Digital Business, and Management Information Technology

I confirm that I have read this submission and believe that I have an appropriate level of expertise to confirm that it is of an acceptable scientific standard.

# Reviewer report: Reviewer response for version 1

**DOI:** 10.5256/f1000research.199216.r487527

**Published:** 2026-06-01

**Authors:** Tony Antonio

**Affiliations:** 1 Universitas Ciputra Surabaya, Surabaya, East Java, Indonesia

1. The authors selected Walumbwa's definition of authentic leadership over Bill George's, but the rationale for this choice was not explicitly stated. One plausible explanation is that Walumbwa's model, which emphasizes high-quality social exchange relationships grounded in trust, fairness, and relational transparency (social exchange theory, SET), is more pertinent to fostering work engagement. Furthermore, this model aligns more closely with the concept of a job resource that enhances psychological safety, meaningfulness, and emotional support, as described in the job demands-resources (JD-R) theory. This alignment offers a clearer justification for its selection.

 2. There is no indication that the paper selection process in the systematic literature review (SLR) explicitly references Walumbwa's definition. Nevertheless, the PRISMA selection process appears to evaluate studies based on the Walumbwa model.

 3. The research question assumes that previous meta-analyses may have produced less accurate pooled effect estimates. A rigorous synthesis using the bivariate Pearson correlation coefficient is needed for a more precise estimate of the population effect size.

 4. Figure 1 identifies authentic leadership as the antecedent variable and work engagement as the outcome. It also highlights several mediating and moderating mechanisms in their relationship.

 However, the authors do not sufficiently support their arguments before introducing this model.

 5. The selection of 1,298 open-access papers could reduce the representation of authentic leadership and work engagement publications because many excellent papers are not open-access. This could be a limitation of this study.

 6. The theoretical review is well written

 7. The meta-analysis and systematic review are well described

 8. The result is well explained

Are the rationale for, and objectives of, the Systematic Review clearly stated?

Yes

Is the statistical analysis and its interpretation appropriate?

Yes

If this is a Living Systematic Review, is the ‘living’ method appropriate and is the search schedule clearly defined and justified? (‘Living Systematic Review’ or a variation of this term should be included in the title.)

Not applicable

Are sufficient details of the methods and analysis provided to allow replication by others?

Yes

Are the conclusions drawn adequately supported by the results presented in the review?

Yes

Reviewer Expertise:

leadership

I confirm that I have read this submission and believe that I have an appropriate level of expertise to confirm that it is of an acceptable scientific standard.
